# Supplementary material for: Osmoregulation in the estuarine diamond-backed terrapin across a broad range of naturally occurring salinities
Source: Biol Open. 2025 Jun 30;14(6):bio062072. doi: 10.1242/bio.062072 (PMC12264734; doi:10.1242/bio.062072)
Supplement: Supplementary information [file biolopen-14-062072-s1.pdf]

Table S1. Environmental, morphological, and blood biochemistry data for individual diamond-backed terrapins (N = 37) used for statistical analysis in this study. T_w = water temperature; SCL = Straight Carapace Length.

Terrapin Code	Date	Season	T_w (°C)	Salinity (psu)	Sex	SCL (mm)	Osmolality (mOsm)	Sodium (mmol·L^{-1})	Chloride (mmol·L^{-1})	Potassium (mmol·L^{-1})	Urea (mmol·L^{-1})	Glucose (mg·dL^{-1})	Total Protein (g·dL^{-1})
CJK	4/29/2021	breeding	25.5	28	F	136	413	149	101	4.4	89.96	81	5.2
BPV	4/29/2021	breeding	21.4	29	M	97	353.5	149	105	4	59.26	94	3
CJL	4/29/2021	breeding	25.5	28	F	119	349	157	107	5.3	30.35	120	3.2
CJM	4/29/2021	breeding	25.5	28	M	103	351.5	156	111	5.3	33.2	137	3.3
BPX	4/29/2021	breeding	21.4	29	M	105	394	157	112	4.2	66.76	149	2.3
CMO	5/13/2021	breeding	18.29	22	M	105	335.5	157	121	3.9	24.28	59	1.8
CNP	5/13/2021	breeding	18.29	22	F	116	334.5	159	120	3.2	18.56	60	2
CPQ	5/20/2021	breeding	25.5	30	F	148	362.5	176	140	4.7	16.42	79	3.7
CPV	5/20/2021	breeding	22.9	30	M	109	385.5	177	138	4.6	33.2	100	2.7
CQV	5/28/2021	breeding	23.8	39	F	151	358	161	128	3.9	40.7	51	2.8
CQX	5/28/2021	breeding	23.8	39	M	105	357	159	129	4.4	49.62	53	2.4
CVX	6/10/2021	breeding	25	26	F	131	319	155	119	3.7	13.92	55	2.5
CVW	6/10/2021	breeding	25	26	M	111	320	153	122	4.1	31.42	83	1.7
HIK	7/13/2021	post-breeding	28	23	M	109	353.5	153	117	3.4	36.77	112	2.5
HJP	7/14/2021	post-breeding	29.2	23	F	151	329.5	156	117	3.7	29.27	55	2.6

Biology Open • Supplementary information

HKL	7/15/2021	post-breeding	29.4	23	M	112	359	158	115	4.3	36.41	148	3.2
HKP	7/20/2021	post-breeding	27.9	21	M	114	341.5	153	110	4.2	33.2	67	3.2
HKO	7/20/2021	post-breeding	27.9	21	M	112	316.5	148	108	4.5	27.13	79	2.8
HKW	7/21/2021	post-breeding	28.7	23	M	112	312	139	110	3.8	26.06	88	1.5
HLO	7/29/2021	post-breeding	28.4	15	F	145	302	165	123	4.8	20.35	75	2.8
HLP	7/29/2021	post-breeding	28.4	15	M	120	264	177	125	4.9	32.49	85	2.5
HLV	8/3/2021	post-breeding	27	13	F	154	312.5	148	107	4.4	13.92	108	2.1
HMO	8/11/2021	post-breeding	28.2	10	M	109	301.5	145	101	4.5	20.71	145	2.3
HNQ	8/12/2021	post-breeding	30	9	F	141	320.5	162	128	4.5	43.91	74	2.5
HMX	8/12/2021	post-breeding	30	9	F	150	306	151	108	3.8	11.78	74	1.9
HPQ	8/31/2021	post-breeding	29.9	21	F	110	331	147	112	4	34.63	67	1.9
HQV	9/8/2021	post-breeding	26.8	29	M	97	343.5	145	109	4.4	34.63	131	2.5
IJV	10/1/2021	post-breeding	26.6	24	F	175	297	147	108	3.8	8.93	83	2.5
JNX	5/18/2022	breeding	22.8	29	M	110	320.5	149	116	3.9	32.84	53	1
JNW	5/18/2022	breeding	22.8	29	M	111	360.5	156	125	3.6	64.97	72	1.2
JQV	5/20/2022	breeding	23.2	33	M	115	326	151	115	5.5	22.13	50	2
JQW	5/20/2022	breeding	23.2	33	F	137	318	150	112	4.4	17.85	67	1.9
KMP	5/25/2022	breeding	28.1	24	F	139	330	178	137	5.1	15.71	71	3.3
KOU	6/15/2022	breeding	27.2	25	F	111	383.5	165	132	4.2	55.34	116	2.5
KQX	6/17/2022	breeding	27.4	27	F	150	355.5	170	139	3.7	16.78	55	1.3

Biology Open • Supplementary information

KVW	7/1/2022	post-breeding	27.8	31	F	152	366.5	170	124	4.9	34.27	77	3.2
KVX	7/1/2022	post-breeding	27.8	31	F	158	380.5	168	131	4.1	41.77	96	2

Biology Open • Supplementary information

```
# Code to perform statistical analyses reported in Pierre et al. 2025
# Osmoregulation in the estuarine diamond-backed terrapin across a broad range of naturally occurring salinities

#=======================================================================

rm(list = ls())

library(dplyr)
library(ggplot2)
library(tidyverse)
library(readr)
library(stats)
library(car)
library(vegan)
library(fitdistrplus)
library(logspline)

#============================================================================

# set the working directory
setwd("")

#IMPORT CSV
#This file contains only individual with Glucose values >50 and <150 (N = 37 terrapins)

read.csv("Pierre et al Biology Open 2025.csv", header=TRUE)
master <- read.csv("Pierre et al Biology Open 2025.csv")

#MAX PRINT if reached
options(max.print = 10000)

dim(master) #tells number of rows and columns
str(master) #structure of the data set

#Convert Sex and Season into factors

master$Sex <- as.factor(master$Sex)
master$Season <- as.factor(master$Season)
class(master$Sex) #double check
class(master$Season)

# Data Exploration

# Basic scatter plot
ggplot(master, aes(x = Salinity, y = Urea)) +
  geom_point(colour = 4)

ggplot(master, aes(x = Salinity, y = Osmolality)) +
  theme_classic(base_size = 20) +
  geom_point(colour = 4) +
  geom_smooth(method = "lm", se = TRUE)
```

```
# ===============================================================================

 # Data exploration to determine if transformation is needed

descdist(master$Urea, discrete = FALSE, boot = 500)

fit.normal <- fitdist(master$Urea, "norm")
plot(fit.normal)

fit.lognormal <- fitdist(master$Urea, "lnorm")
plot(fit.lognormal)

#compare fits for different distributions
fit.normal$aic
fit.lognormal$aic

fit.metrics <- lapply(ls(pattern = "fit\\."), function(variable) {
 fit = get(variable, envir = .GlobalEnv)
 with(fit, data.frame(name = variable, aic, loglik))
})
do.call(rbind, fit.metrics)

# ===============================================================================

#NMDS #NMDS #NMDS (https://jkzorz.github.io/2020/04/04/NMDS-extras.html)

# Additional descriptions (https://uw.pressbooks.pub/appliedmultivariatestatistics/chapter/nmds/)

#Data Contains Both Continuous and Categorical data
#sex and season = categorical
#salinity, Tw, and SCL continuous

#BLOOD & ENVIRONMENTAL VARIABLES

# environmental variables = "environment", blood variables = "community"
# blood is equivalent to "comm" - a community data matrix (n sample units (i.e. terrapins), p species (blood
variables))
# ================================================================================

#blood variables data set

blood <- master %>% dplyr::select(Osmolality,Sodium,Chloride,Potassium,Glucose,Urea,TotalProtein)
str(blood)

# =================================================================================

#environmental factors data set

env <- master %>% dplyr::select(Salinity,Tw,SCL,Sex, Season)
str(env)
```

Biology Open • Supplementary information

```
# ==============================================================================

# convert blood variables to a matrix
##In R, a matrix is a collection of elements of the same data type
#(numeric, character, or logical) arranged into a fixed number of rows and columns.

m_blood= as.matrix(blood)

# ==============================================================================

# Perform the NMDS ordination
set.seed(123)
nmds=metaMDS(m_blood, distance = "bray")
nmds  #stress=0.1307215 (<0.2 is good/ok - closer to 1 is better)

#explore the effects of individual points
gof <- goodness(object = nmds)
plot(nmds, display = "sites", type = "none")
points(nmds, display = "sites", cex = 2*gof/mean(gof))

#Shepard Plot to evaluate the fit of the NMDS ordination
#assessed by plotting the original dissimilarities (z$diss) against the (Euclidean) ordination distances (z$dist)
plot(nmds$diss, nmds$dist)

#calculate the distances directly from the data to verify that the mapped values in the Shepard plot
#are from the original data and the coordinates in the ordination space
plot(vegdist(blood), dist(scores(nmds, "sites")))

#Shepard plots can also be created using the stressplot() function in vegan
#Preferred method - it provides R2 values (results below for NMDS without Alb and Glb)
stressplot(nmds)  #non-metric fit R2 = 0.983, linear fit R2 = 0.922

#Coordinates
nmds$points %>% head()
#These coordinates are the positions of each sample unit in the ordination space.
#Points near one another are similar in their multivariate response

#save them to their own object:
nmds.points <- data.frame(nmds$points)

#Initial ordination plot using coordinates of the points:
p <- ggplot(data = nmds.points, aes(x = MDS1, y = MDS2)) +
 theme_bw() +
 theme(axis.title = element_blank(),
      axis.ticks = element_blank(),
      axis.text = element_blank())
p + geom_point()

#plot final configuration of metaMDS results
plot(nmds, display = "sites")
```

Biology Open • Supplementary information

```
#sites = individual terrapin, species = blood variables?
#indicate where a given blood variable falls out
sp <- wascores(x = nmds$points, w = blood, expand = TRUE)
ions <- data.frame(sp[c("Osmolality","Sodium","Chloride","Potassium","Glucose","Urea","TotalProtein"), ])

p +
  geom_point() +
  geom_text(data = ions, label = rownames(ions))

# ==============================================================================

# run the envfit function with our environmental data frame, env.
en = envfit(nmds, env, permutations = 999, na.rm = TRUE)
en

# ================================================================================

# Plot both the NMDS and environmental data
# (Longer arrows, mean a stronger association.)
plot(nmds)
plot(en)
# The envfit vectors and factors (blue) are overlaid on the original NMDS plot
# with individual terrapins as black circles and blood variables as red crosses.

# ==============================================================================
#extract NMDS scores (x and y coordinates) for sites from newer versions of
#vegan package
data.scores = as.data.frame(scores(nmds)$sites)

#add columns to data frame
data.scores$Season = master$Season
data.scores$Salinity = master$Salinity
data.scores$Sex = master$Sex
data.scores$Tw = master$Tw
data.scores$SCL = master$SCL

head(data.scores)

# ==============================================================================

# Extracting the required information from the envfit result
# The envfit output contains information on the length of the segments for each variable
# environmental variables with a longer segment are more strongly correlated with the data
# Because my data contained continuous and categorical environmental variables,
# I'm extracting the information from both separately using the "vectors" and "factors" options respectively.
en_coord_cont = as.data.frame(scores(en, "vectors")) * ordiArrowMul(en)
en_coord_cat = as.data.frame(scores(en, "factors")) * ordiArrowMul(en)

# ==============================================================================
#GG Plot
```

Biology Open • Supplementary information

```
#Breeding vs Non-breeding
gg_season = ggplot(data = data.scores, aes(x = NMDS1, y = NMDS2)) +
 geom_point(data = data.scores, aes(colour = Season, shape = Sex), size = 3, alpha = 0.5) +
 scale_colour_manual(values = c("red", "blue")) +
 theme(axis.title = element_text(size = 10, face = "bold", colour = "grey30"),
    panel.background = element_blank(), panel.border = element_rect(fill = NA, colour = "grey30"),
    axis.ticks = element_blank(), axis.text = element_blank(), legend.key = element_blank(),
    legend.title = element_text(size = 10, face = "bold", colour = "grey30"),
    legend.text = element_text(size = 9, colour = "grey30")) +
 labs(colour = "Season") +
 labs(shape = "Sex")

gg_season

# And below is the code for the NMDS plot and the envfit data:
gg_season_upd = ggplot(data = data.scores, aes(x = NMDS1, y = NMDS2)) +
 geom_point(data = data.scores, aes(colour = Season, shape = Sex), size = 3, alpha = 0.5) +
 scale_colour_manual(values = c("red", "blue"))  +
 geom_segment(aes(x = 0, y = 0, xend = NMDS1, yend = NMDS2),
       data = en_coord_cont, size =1, alpha = 0.5, colour = "grey30") +
 geom_point(data = en_coord_cat, aes(x = NMDS1, y = NMDS2),
       shape = "diamond", size = 4, alpha = 0.6, colour = "navy") +
 geom_text(data = en_coord_cat, aes(x = NMDS1, y = NMDS2+0.04),
      label = row.names(en_coord_cat), colour = "navy", fontface = "bold") +
 geom_text(data = en_coord_cont, aes(x = NMDS1, y = NMDS2), colour = "grey30",
      fontface = "bold", label = row.names(en_coord_cont)) +
 theme(axis.title = element_text(size = 10, face = "bold", colour = "grey30"),
    panel.background = element_blank(), panel.border = element_rect(fill = NA, colour = "grey30"),
    axis.ticks = element_blank(), axis.text = element_blank(), legend.key = element_blank(),
    legend.title = element_text(size = 10, face = "bold", colour = "grey30"),
    legend.text = element_text(size = 9, colour = "grey30")) +
 labs(colour = "Season")

gg_season_upd

# Here is code WITHOUT points and text for the categorical variables
gg_season_upd = ggplot(data = data.scores, aes(x = NMDS1, y = NMDS2)) +
 geom_point(data = data.scores, aes(colour = Season, shape = Sex), size = 3, alpha = 0.5) +
 scale_colour_manual(values = c("red", "blue"))  +
 geom_segment(aes(x = 0, y = 0, xend = NMDS1, yend = NMDS2),
       data = en_coord_cont, size =1, alpha = 0.5, colour = "grey30") +
 geom_text(data = en_coord_cont, aes(x = NMDS1, y = NMDS2), colour = "grey30",
      fontface = "bold", label = row.names(en_coord_cont)) +

 theme(axis.title = element_text(size = 10, face = "bold", colour = "grey30"),
    panel.background = element_blank(), panel.border = element_rect(fill = NA, colour = "grey30"),
    axis.ticks = element_blank(), axis.text = element_blank(), legend.key = element_blank(),
    legend.title = element_text(size = 10, face = "bold", colour = "grey30"),
    legend.text = element_text(size = 9, colour = "grey30")) +
 labs(colour = "Season")
```

Biology Open • Supplementary information

```
gg_season_upd

#having trouble overlaying the species (i.e. blood variables) because code has MDS1 and MDS2 as axes
#instead of NMDS1 and NMDS2
geom_point(data = nmds.points)
geom_text(data = variables, label = rownames(variables))

# ==================================================================================

#blood is equivalent to "comm" - a community data matrix (n sample units (i.e. terrapins), p species (blood
variables))
m_blood= as.matrix(blood)

#Perform the NMDS ordination
set.seed(123)
nmds=metaMDS(m_blood, distance = "bray")
nmds  #stress=0.1182361  (# <0.2 is good/ok)

# run the NMDS using the metaMDS() function, and plot the resulting object:
# THIS IS THE CODE THAT GIVES GRAPH WITH SPECIES (i.e. BLOOD VARIABLES)
sol <- metaMDS(blood)
plot(sol, display = c("sites", "species"), type = "t")

##Check the goodness of fit of the visual ordination procedure
sol$stress
stressplot(sol)

# ==================================================================================
# Testing significant difference of multivariate data across variables - permutational test
# PERMANOVA background information - https://uw.pressbooks.pub/appliedmultivariatestatistics/?s=permanova

adonis2(formula = blood ~ Salinity, data = env)

adonis2(formula = blood ~ Tw, data = env)

adonis2(formula = blood ~ SCL, data = env)

adonis2(formula = blood ~ Sex, data = env)

adonis2(formula = blood ~ Season, data = env)

# ================================================================================

### Linear Models! ###

# standardize continuous covariates
# standardization/normalization is important when continuous variables are measured at different scales
# it allows you to rescale variables to have equal range and/or variance so one is not weighted more than another
master$Salinity.c <- (master$Salinity - mean(master$Salinity))/sd(master$Salinity)
master$Tw.c <- (master$Tw - mean(master$Tw))/sd(master$Tw)
master$SCL.c <- (master$SCL - mean(master$SCL))/sd(master$SCL)
```

Biology Open • Supplementary information

```
# log transform the response variables
master$Osmolality.log <- log(master$Osmolality + 1)
master$Sodium.log <- log(master$Sodium + 1)
master$Chloride.log <- log(master$Chloride + 1)
master$Potassium.log <- log(master$Potassium + 1)
master$Urea.log <- log(master$Urea + 1)
master$Glucose.log <- log(master$Glucose + 1)
master$TotalProtein.log <- log(master$TotalProtein + 1)

# plot histogram to check distribution
hist(master$Osmolality.log)
hist(master$Sodium.log)
hist(master$Chloride.log)
hist(master$Potassium.log)
hist(master$Urea.log)
hist(master$Glucose.log)
hist(master$TotalProtein.log)

# QQplot and QQline
# helps to visualize normality through Q-Q plot
qqnorm(master$Osmolality.log); qqline(master$Osmolality.log, col = 2, lwd = 2, lty = 2)

# run linear models on the log-transformed data
# explanatory variables include Salinity, Tw, and SCL based on NMDS and PERMANOVA results
# Get 95% confidence intervals for model coefficients

Osmolality.model <- lm(Osmolality.log ~ Salinity.c + SCL.c + Season, data = master)
summary (Osmolality.model)
conf_intervals <- confint(Osmolality.model)
print(conf_intervals)

Sodium.model <- lm(Sodium.log ~ Salinity.c + SCL.c + Season, data = master)
summary (Sodium.model)
conf_intervals <- confint(Sodium.model)
print(conf_intervals)

Chloride.model <- lm(Chloride.log ~ Salinity.c + SCL.c + Season, data = master)
summary (Chloride.model)
conf_intervals <- confint(Chloride.model)
print(conf_intervals)

Potassium.model <- lm(Potassium.log ~ Salinity.c + SCL.c + Season, data = master)
summary (Potassium.model)
conf_intervals <- confint(Potassium.model)
print(conf_intervals)

Urea.model <- lm(Urea.log ~ Salinity.c + SCL.c + Season, data = master)
summary (Urea.model)
conf_intervals <- confint(Urea.model)
print(conf_intervals)
```

Biology Open • Supplementary information

```
Glucose.model <- lm(Glucose.log ~ Salinity.c + SCL.c + Season, data = master)
summary (Glucose.model)
conf_intervals <- confint(Glucose.model)
print(conf_intervals)

TotalProtein.model <- lm(TotalProtein.log ~ Salinity.c + SCL.c + Season, data = master)
summary (TotalProtein.model)
conf_intervals <- confint(TotalProtein.model)
print(conf_intervals)

# ===============================================================================

# GRAPHS to illustrate significant effects

# Plot raw data (not transformed) to show trends with significant variables

# Salinity has a significant positive effect on Osmolality and Urea
ggplot(master, aes(x = Salinity, y = Osmolality)) +
 theme_classic(base_size = 14) +
 theme(text=element_text(family="Times New Roman")) +
 geom_point(size = 3) +        #black dots if color left blank
 labs(x = "Salinity (psu)",
    y = "Osmolality (mOsm)")

ggplot(master, aes(x = Salinity, y = Urea)) +
 theme_classic(base_size = 14) +
 theme(text=element_text(family="Times New Roman")) +
 geom_point(size = 3) +
 labs(x = "Salinity (psu)",
     y = "Urea (mmol L-1)")


# SCL has an inverse relationship with Urea and Glucose

ggplot(master, aes(x = SCL, y = Urea)) +
 theme_classic(base_size = 14) +
 theme(text=element_text(family="Times New Roman")) +
 geom_point(size = 3) +        #black dots if color left blank
 labs(x = "Straight Carapace Length (mm)",
    y = "Urea (mmol L-1)")

ggplot(master, aes(x = SCL, y = Glucose)) +
 theme_classic(base_size = 14) +
 theme(text=element_text(family="Times New Roman")) +
 geom_point(size = 3) +
 labs(x = "Straight Carapace Length (mm)",
    y = "Glucose (mg dL-1)")


# Plot model outputs with confidence intervals (log-transformed and standardized data)
```

Biology Open • Supplementary information

```
# Salinity has a significant positive effect on Osmolality and Urea

ggplot(master, aes(x = Salinity.c, y = Osmolality.log)) +
  theme_classic(base_size = 14) +
  theme(text=element_text(family="Times New Roman")) +
  geom_point(size = 3) +
  labs(x = "Salinity (standardized)",
     y = "log Osmolality") +
  geom_smooth(method = "lm", se = TRUE)

ggplot(master, aes(x = Salinity.c, y = Urea.log)) +
  theme_classic(base_size = 14) +
  theme(text=element_text(family="Times New Roman")) +
  geom_point(size = 3) +
  labs(x = "Salinity (standardized)",
     y = "log Urea") +
  geom_smooth(method = "lm", se = TRUE)


# SCL has an inverse relationship with Urea and Glucose

ggplot(master, aes(x = SCL.c, y = Urea.log)) +
  theme_classic(base_size = 14) +
  theme(text=element_text(family="Times New Roman")) +
  geom_point(size = 3) +
  labs(x = "Straight Carapace Length (standardized)",
     y = "log Urea") +
  geom_smooth(method = "lm", se = TRUE)

ggplot(master, aes(x = SCL.c, y = Glucose.log)) +
  theme_classic(base_size = 14) +
  theme(text=element_text(family="Times New Roman")) +
  geom_point(size = 3) +
  labs(x = "Straight Carapace Length (standardized)",
     y = "log Glucose") +
  geom_smooth(method = "lm", se = TRUE)
```

Dataset 1.

Available for download at
https://journals.biologists.com/bio/article-lookup/doi/10.1242/bio.062072#supplementary-data
